# MRI-Based Deep Learning Method for Classification of IDH Mutation Status

**DOI:** 10.3390/bioengineering10091045

**Published:** 2023-09-05

**Authors:** Chandan Ganesh Bangalore Yogananda, Benjamin C. Wagner, Nghi C. D. Truong, James M. Holcomb, Divya D. Reddy, Niloufar Saadat, Kimmo J. Hatanpaa, Toral R. Patel, Baowei Fei, Matthew D. Lee, Rajan Jain, Richard J. Bruce, Marco C. Pinho, Ananth J. Madhuranthakam, Joseph A. Maldjian

**Affiliations:** 1 Department of Radiology, University of Texas Southwestern Medical Center, Dallas, TX 75390, USA; ben.wagner@utsouthwestern.edu (B.C.W.); nghi.truong@utsouthwestern.edu (N.C.D.T.); james.holcomb@utsouthwestern.edu (J.M.H.); divya.reddy@utsouthwestern.edu (D.D.R.); niloufar.saadat@utsouthwestern.edu (N.S.); bfei@utdallas.edu (B.F.); marco.pinho@utsouthwestern.edu (M.C.P.); ananth.madhuranthakam@utsouthwestern.edu (A.J.M.); joseph.maldjian@utsouthwestern.edu (J.A.M.); 2 Department of Pathology, University of Texas Southwestern Medical Center, Dallas, TX 75390, USA; kimmo.hatanpaa@utsouthwestern.edu; 3 Department of Neurological Surgery, University of Texas Southwestern Medical Center, Dallas, TX 75390, USA; toral.patel@utsouthwestern.edu; 4 Department of Bioengineering, University of Texas at Dallas, Richardson, TX 75080, USA; 5 Department of Radiology, NYU Grossman School of Medicine, New York, NY 10016, USA; matthew.lee4@nyulangone.org (M.D.L.); rajan.jain@nyulangone.org (R.J.); 6 Department of Neurosurgery, NYU Grossman School of Medicine, New York, NY 10016, USA; 7 Department of Radiology, University of Wisconsin School of Medicine and Public Health, Madison, WI 53726, USA; rbruce@uwhealth.org

**Keywords:** nnU-Net, deep learning, IDH, U-net, brain tumor, MRI, CNN, gliomas

## Abstract

Isocitrate dehydrogenase (IDH) mutation status has emerged as an important prognostic marker in gliomas. This study sought to develop deep learning networks for non-invasive IDH classification using T2w MR images while comparing their performance to a multi-contrast network. Methods: Multi-contrast brain tumor MRI and genomic data were obtained from The Cancer Imaging Archive (TCIA) and The Erasmus Glioma Database (EGD). Two separate 2D networks were developed using *nnU-Net*, a T2w-image-only network (T2-net) and a multi-contrast network (MC-net). Each network was separately trained using TCIA (227 subjects) or TCIA + EGD data (683 subjects combined). The networks were trained to classify IDH mutation status and implement single-label tumor segmentation simultaneously. The trained networks were tested on over 1100 held-out datasets including 360 cases from UT Southwestern Medical Center, 136 cases from New York University, 175 cases from the University of Wisconsin–Madison, 456 cases from EGD (for the TCIA-trained network), and 495 cases from the University of California, San Francisco public database. A receiver operating characteristic curve (ROC) was drawn to calculate the AUC value to determine classifier performance. Results: T2-net trained on TCIA and TCIA + EGD datasets achieved an overall accuracy of 85.4% and 87.6% with AUCs of 0.86 and 0.89, respectively. MC-net trained on TCIA and TCIA + EGD datasets achieved an overall accuracy of 91.0% and 92.8% with AUCs of 0.94 and 0.96, respectively. We developed reliable, high-performing deep learning algorithms for IDH classification using both a T2-image-only and a multi-contrast approach. The networks were tested on more than 1100 subjects from diverse databases, making this the largest study on image-based IDH classification to date.

## 1. Introduction

The World Health Organization (WHO) revised glioma classification in 2016 with the observation that tumors with isocitrate dehydrogenase (IDH) mutation have a better prognosis than those with wildtype IDH [1]. IDH-mutated tumors also have different diagnostic and therapy responses than wildtype tumors. As a result, different diagnostic and therapeutic approaches are necessary for IDH-mutated and wildtype gliomas. Currently, the only way to conclusively detect an IDH-mutated glioma involves immunohistochemistry or gene sequencing on a tissue sample obtained either via a biopsy or surgical removal. However, information from the TCGA indicates that a mere 35% of biopsy samples have sufficient tumor content to allow for accurate molecular characterization [2]. Therefore, developing a robust and reliable non-invasive approach would be beneficial for these patients.

MR spectroscopy has the potential to identify IDH mutations. The mutant IDH enzyme produces the oncometabolite 2-HG [3]. MR spectroscopic methods have been developed to detect 2-HG non-invasively in brain tumors [4,5,6,7]. However, in a clinical setting, the spectral imaging data can be difficult to interpret due to artifacts, patient motion, poor shimming, small voxel sizes, non-ideal tumor location, or the presence of hemorrhage or calcification. Even when the spectra are of good quality, the false-positive rate of this technique is over 20%, making reliable clinical implementation difficult [8].

IDH wildtype gliomas are typically treated with more aggressive regimens than IDH mutant gliomas. Specific chemotherapeutic interventions are more effective in IDH-mutated gliomas (e.g., temozolomide) [9,10,11,12,13]. Furthermore, surgical resection of non-enhancing tumor volume in grade 3–4 IDH-mutated tumors has been shown to improve survival [14]. However, the determination of IDH mutation status is currently achieved through direct tissue sampling, which can be difficult to achieve. The amount of tumor tissue in biopsy samples may not always be sufficient for a complete molecular characterization [2]. Therefore, developing a robust and reliable non-invasive approach would be beneficial for these patients.

Recent research has demonstrated that deep learning is more effective than conventional machine learning techniques for predicting the genetic and molecular biology of tumors when using MRI [15,16,17]. However, these techniques are not yet suitable for clinical use as they rely on labor-intensive tumor pre-segmentation, significant pre-processing, or the use of multi-contrast acquisitions that frequently get disrupted by patient movement during lengthy scan times. Furthermore, the existing approaches utilize a 2D (slice-wise) classification method known to be susceptible to *data leakage problems* [18,19]. Two-dimensional slice-wise models, when applied to cross-sectional imaging data, are specifically prone to data leakage as randomized data division might happen across all subjects. This can lead to the creation of training, validation, and test sets where neighboring slices from the same subject are found in distinct data subsets. Since these adjacent slices often contain a great deal of shared information, this method can artificially enhance accuracy levels by introducing bias into the testing phase. Our own work with 2D IDH models has shown a boost of over 6% in classification accuracy when data leakage is permitted [20]. Another limitation of these previous studies is their lack of evaluation of large, true external held-out datasets, limiting the generalizability of their results.

This study sought to develop highly accurate, fully automated, and reliable deep learning algorithms for IDH classification. This study’s evaluation of large, diverse, and true external held-out datasets represents an important milestone in the journey towards clinical translation.

## 2. Materials and Methods

### 2.1. Datasets

#### 2.1.1. Training Data

Multi-parametric brain MRI data and genomic information of glioma patients were obtained from the publicly available TCIA (The Cancer Imaging Archive)/TCGA (The Cancer Genome Atlas) databases and The Erasmus Glioma Database (EGD) [21,22,23]. Subjects were screened for the availability of IDH mutation status and multi-contrast MR images. Only pre-operative studies were included in the final dataset. The TCIA database consists of 227 subjects (94 IDH mutated, 133 IDH wildtype), and the EGD database consists of 456 subjects (150 IDH mutated, 306 IDH wildtype). The combination of TCIA and EGD provided 683 subjects with multi-contrast MR images and ground truth IDH status. Ground truth IDH status of the TCIA and EGD datasets was determined using the gold-standard Sanger method and next-generation sequencing, respectively [22,24,25,26]. The subject IDs used for training and their corresponding IDH statuses are listed in Table S1 of the supplementary data.

#### 2.1.2. Testing Data

External testing data were obtained from 5 institutions, including 2 publicly available datasets. The public data were obtained from the University of California, San Francisco (UCSF) Preoperative Diffuse Glioma MRI dataset [27] with 495 subjects and the previously mentioned EGD [22] with 456 subjects. The collaborator/in-house datasets were obtained from three institutions, the UT Southwestern Medical Center (UTSW) with 360 subjects, New York University (NYU) with 136 subjects, and the University of Wisconsin–Madison (UWM) with 175 subjects (Table 1). Subjects were screened for the availability of IDH mutation status and multi-contrast MR images. Only pre-operative studies were included in the final dataset. IDH mutation status was determined either by immunohistochemistry or next-generation sequencing when available.

### 2.2. Pre-Processing

Multi-contrast native space images, including pre-contrast and post-contrast T1-weighted, T2-weighted, and T2-weighted fluid attenuation inversion recovery (FLAIR), from TCIA, UTSW, NYU, and UWM were pre-processed using the FeTS platform (version 002) [28], which co-registers the images, brings them to SRI24 template space [29], and performs skull-stripping and multi-class brain tumor segmentation. The UCSF data were already pre-processed (co-registered in SRI24 template space and skull-stripped) [27]. The EGD data were also pre-processed (co-registered to MNI152 space), with the data skull-stripped using Advanced Normalization Tools (ANTS) [22,30]. Additionally, all datasets were (a) N4 bias corrected and (b) intensity normalized to zero-mean and unit variance [31,32].

**Figure 1:**
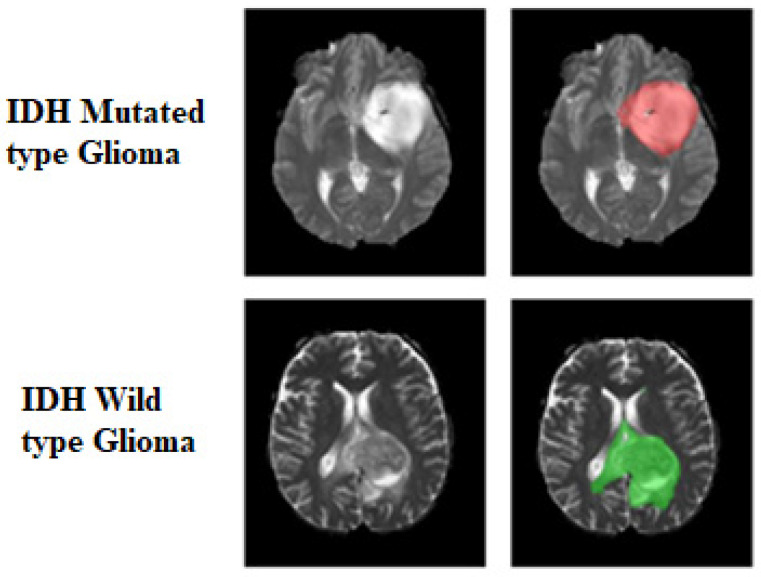
Ground truth tumor masks. The green voxels represent IDH wildtype (values of 2). The red voxels represent IDH mutated (values of 1). The ground truth labels have the same mutation status for all voxels in each tumor.

Multi-label FeTS tumor segmentations were combined to generate the whole-tumor masks. The ground truth tumor masks in IDH-mutated cases were marked with 1s, while the ground truth tumor masks for IDH wildtype were labeled with 2s (Figure 1). These tumor masks were used as the ground truth for tumor segmentation in the training step.

### 2.3. Network Details

**Figure 2:**
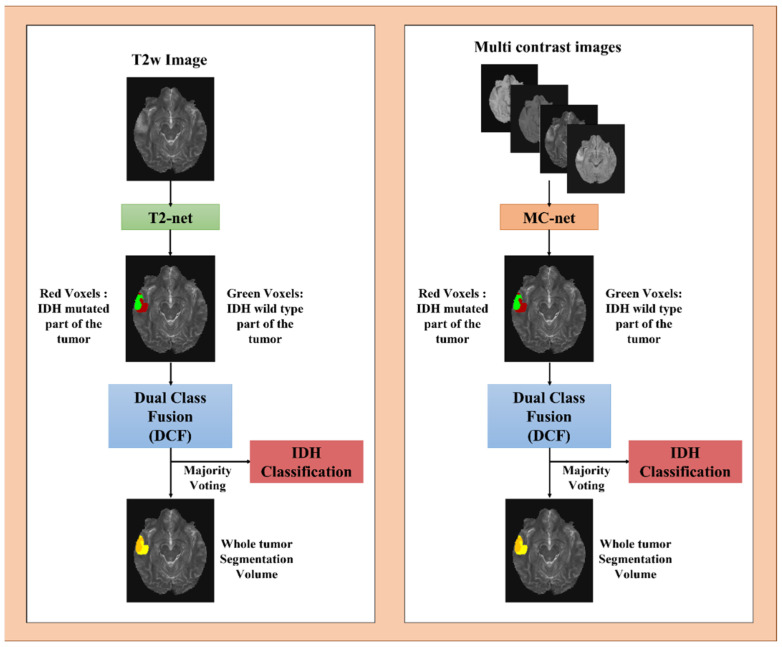
Overview of voxel-wise classification of IDH mutation status. Volumes were combined through dual-class fusion to remove false positives and create a tumor segmentation volume. Majority voting was applied across the voxels to predict the overall IDH mutation status.

Two separate 2D networks were designed using the *nnU-Net* package. These were a T2w-image-only network (T2-net) and a multi-contrast network (MC-net) (Figure 2). Each network was trained on TCIA data only (227 subjects) and a combination of TCIA + EGD datasets (683 subjects combined) separately. The *nnU-Net’s* trainer class *nnU-NetTrainerV2_DA5*, representing advanced data augmentation steps, was used. The *nnU-Net*’s inbuilt five-fold cross-validation (CV) training-only procedure was implemented for each network. Generic nnU-Nets were trained to perform voxel-wise dual-class segmentation of the whole tumor, with Class 1 representing IDH mutated and Class 2 representing IDH wildtype. Generic *nnU-Nets* were selected as they reuse all the generated feature maps, such that that each layer in the design has a direct supervision signal [33].

### 2.4. Network Implementation and Cross-Validation

The networks were implemented using PyTorch [34] and *nnU-Net* packages [35]. To generalize the performance of the networks, *nnU-Net’s* inbuilt cross-validation (CV) training-only procedure was utilized. A 5-fold CV procedure was implemented for training each network. The subjects were randomly shuffled, and 80% of the data were used for training, and 20% of the data were used for in-training validation (model optimization). Data shuffling and splitting were implemented at the subject level to avoid the problem of *data leakage* [18,19]. During each fold of the cross-validation procedure, the subjects were reshuffled to generate the training and in-training validation datasets. It is important to note that each fold in the cross-validation process signifies a new training phase with a unique combination of the dataset. The in-training validation set aids in enhancing network performance throughout training. The in-training validation dataset is used by the algorithm to assess its performance after every training cycle and to update the model’s hyperparameters (as detailed in Supplementary Table S2). It is not considered a true held-out dataset since the algorithm modifies its performance based on the results from the in-training validation dataset during each round. Once the algorithm has finished all the training folds, it is then evaluated on the actual held-out external datasets to gauge its performance.

### 2.5. Testing Procedure

Testing data included all the external datasets for the TCIA-trained networks (EGD, UTSW, NYU, UWM, and UCSF), representing a total of 1622 subjects, and all external datasets without the EGD dataset for the TCIA + EGD-trained networks (total of 1166 subjects). Output segmentations of the five trained models from each fold of the CV procedure were ensembled to obtain the final two-class segmentation volume. A dual-class fusion (DCF) technique was developed to merge the two-class segmentation. The two classes were combined, and the largest connected component was extracted using a 3D connected component algorithm in MATLAB(R). This combination of classes yielded a whole-tumor segmentation map (Figure 2). Majority voting over the voxel-wise classes resulted in an IDH classification for each subject. The networks were run on Tesla A100 NVIDIA GPUs, and the developed IDH classification procedure was completely automated. A tumor segmentation map is a natural output of the voxel-wise classification approach.

### 2.6. Statistical Analysis

The performance of both T2-net and MC-net was statistically analyzed using MATLAB (2019a) and R programming 2022. The accuracy of these two networks was determined by a majority-voting process with a voxel-wise cutoff of 50%. This threshold was then utilized to compute the subject-level accuracy, sensitivity, and specificity. A receiver operating characteristic curve (ROC curve) was also calculated for the ensembled results of each combination of the network, training approach, and trainer class. The ROC methodology can be found in the supplementary data. The dice similarity coefficient was used to compare the spatial overlap between the network’s segmentation and the ground truth.

## 3. Results

### 3.1. T2-Net

Table 2 represents the testing results for T2-net. The network trained on a combined dataset of TCIA + EGD achieved the best accuracies across all institutions with an overall accuracy of 87.6% and an overall AUC of 0.8931. It also achieved a mean dice score as high as 0.85 ± 0.15.

### 3.2. MC-Net

Table 3 represents the testing results for MC-net. The network trained on the combined dataset of TCIA + EGD achieved the best accuracies across all institutions with an overall accuracy of 92.8% and an overall AUC of 0.9646 and outperformed the T2-only network. The MC-net achieved a mean dice score as high as 0.89 ± 0.13.

### 3.3. ROC Analysis

**Figure 3:**
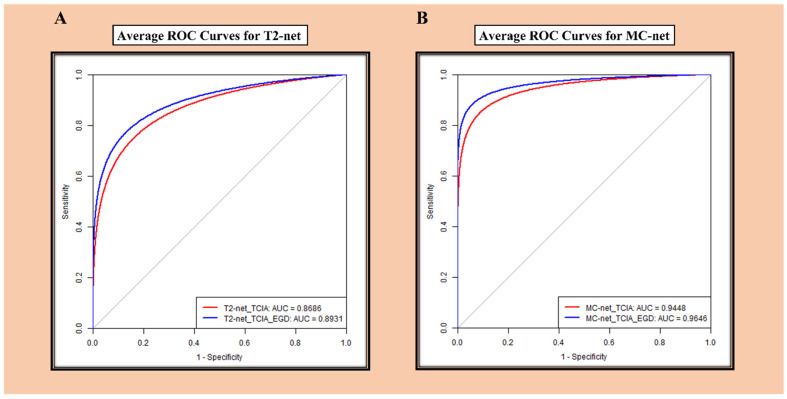
(**A**) ROC analysis for T2-net. (**B**) ROC analysis for MC-net.

ROC curves for the average accuracy of T2-net and MC-net are provided in Figure 3. MC-net demonstrated better performance than T2-net.

### 3.4. Voxel-Wise Classification

**Figure 4:**
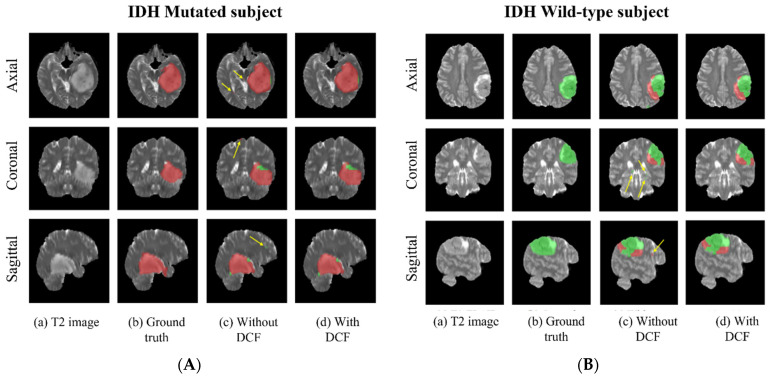
(**A**) Example voxel-wise segmentation for an IDH-mutated and IDH-wildtype tumor. T2 image (**a**). Ground truth segmentation (**b**). Voxel-wise predictions without DCF (**c**) and after DCF (**d**). Yellow arrows in indicate false positives. Red voxels depict IDH-mutated class, and green voxels depict IDH wildtype (**B**).

Since these networks are voxel-wise classifiers, they provide simultaneous tumor segmentation. Figure 4A,B shows examples of the voxel-wise classification for an IDH-wildtype and an IDH-mutated case using the T2-net. The DCF procedure successfully eliminated false positives. The average voxel-wise accuracy for each network and mutation status was also calculated. In general, all the models exhibited higher voxel-wise accuracies for the wildtype class compared to for the mutated class (Table 4).

### 3.5. Training and Segmentation Times

Each cross-validation procedure took approximately 5 days to train. The trained networks took only one minute to segment the whole tumor, implement DCF, and predict the IDH mutation status for each subject.

## 4. Discussion

We developed and evaluated MRI-based deep learning algorithms for IDH classification in gliomas. Two separate 2D networks were developed using the *nnU-Net* package, a T2w-image-only network (T2-net) and a multi-contrast network (MC-net). The performance of T2-net and MC-net was compared across different datasets, revealing the potential of T2-net for future improvements and the advantages of our networks over existing IDH classification algorithms.

The overall accuracy of the T2-net trained on the combined dataset of TCIA + EGD was 87.6%, with an AUC of 0.8931, while the MC-net trained on the same dataset achieved an overall accuracy of 92.8% and an AUC of 0.9646. The MC-net outperformed the T2-net across all institutions, suggesting that using multi-contrast MR images enhances the performance of the algorithm (Table 3 and Table 4).

Despite the superior performance of MC-net, the T2-net demonstrated a high overall accuracy and AUC, indicating the potential of T2-net in future applications. Our results suggest that the use of T2w images has the potential to achieve similar performance as multi-contrast images. The T2-net’s performance can be improved by incorporating additional data, refining the network architecture, and optimizing hyperparameters.

The ability to use only T2-weighted images offers several advantages over multi-contrast MR images. T2w MR images are widely available and are a part of routine clinical practice, making them more accessible and practical in a clinical setting. This accessibility ensures that the T2-net can be applied to a larger patient population. A T2w MR sequence requires shorter acquisition times compared to multi-contrast MR images, reducing the likelihood of motion artifacts and improving patient comfort. Relying on a single MR image simplifies the pre-processing and prediction pipeline, accelerating the implementation of T2-net in clinical setting, where acquiring multi-contrast MR images may not always be possible. The time taken by T2-net to segment the entire tumor, execute the DCF procedure, and predict the IDH mutation status for a subject is under one minute. All these advantages of T2-net make clinical translation much more straightforward.

Our T2-net and MC-net exhibited excellent performance in comparison to several existing machine learning algorithms. Zhang et al. achieved 80% IDH classification accuracy using a radiomics approach with an SVM model and multi-modal MRI features [15] and 86% classification accuracy employing multi-modal MRI, clinical features, and a random forest algorithm [16]. Zhou et al. reached an AUC of 0.91 by incorporating MR feature extraction and a random forest algorithm [36]. Li et al. attained an AUC of 0.95 by combining deep-learning-based radiomics (DLR) on multi-modal MR images [37]. Choi et al. developed a complex hybrid approach based on 2D tumor images, radiomic features, 3D tumor shape, and loci guided by a tumor segmentation CNN, yet only achieved 78% IDH classification accuracy on the TCIA dataset [38]. Matsui et al. developed a multi-modality model to diagnose the molecular subtype and to predict the three-group classification directly, achieving a classification accuracy of only 68.7% for the test dataset [39]. Karami et al. developed DL-based models using conventional MRI and multi-shell diffusion MRI to achieve a classification accuracy of 75% [40]. Pasquini et al. developed a convolutional neural network (CNN) on multiparametric MRI, achieving a classification accuracy of 67% using T2w images alone [41]. In contrast, our T2-net reached an overall IDH classification accuracy of 87.5%, while the MC-net attained an overall IDH classification accuracy of 92.7%. Our networks demonstrated competitive performance when compared to the algorithm developed by Chang et al. (accuracy of 94%) using the TCIA database [17]. However, Chang et al.’s algorithm uses a 2D (slice-wise) classification approach, which is prone to *data leakage problems* [17]. In contrast, our algorithms employ subject-level data splitting, which mitigates the data leakage issue commonly found in slice-wise methodologies [18,19]. Our work also overcomes the limitations of previous studies by assessing the performance on large, true external held-out datasets, yielding more reliable accuracy estimates.

Several factors may contribute to the superior performance of our networks compared to previously published machine learning algorithms. The primary reason is the employment of simple *nnU-Nets*, in contrast to the hybrid and more complex networks reported earlier. Simple UNets are easy to use and train, with excellent generalizability and interpretability and a reduced risk of overfitting [42]. Additionally, the *nnU-Nets* architecture is advantageous as it automatically configures itself, including pre-processing, network architecture, training, and post-processing [35]. The *nnU-Nets* carry information from each preceding layer to the subsequent ones, making these networks more trainable and helping to minimize overfitting [35,43]. The *nnU-Nets* were designed to leverage information from MR images and differentiate imaging features between brain tumors and artifacts and are equipped to handle variations in tumor location, size, and appearance using our pre-processing and data augmentation strategies. It is worth noting that approximately 10% of the TCIA dataset used for training had some kind of visible motion or artifact. Despite these complexities, the network was able to effectively learn and generalize, indicating its ability to handle such challenges. The dual-class fusion (DCF) stage also aids in effectively discarding false positives and enhances segmentation accuracy by omitting unrelated voxels that are not connected to the tumor. Additionally, the networks require minimal pre-processing and do not necessitate the extraction of pre-engineered features from the images or histopathological data [44].

The trained *nnU-Nets* are voxel-wise classifiers, giving a classification for each individual voxel in the image. This provides simultaneous single-label tumor segmentation (e.g., the combination of voxels classified as IDH mutated and non-mutated forms the overall tumor segmentation). These networks showed impressive performance in whole-tumor segmentation, with T2-net yielding dice coefficients of 0.85 and the multi-contrast MC-net yielding 0.89. These coefficients align with top performers from various BraTS tumor segmentation challenges [43,45,46,47].

These voxel-wise classifiers lead to classifications of parts of each tumor as either IDH mutated or IDH wildtype. This reflects the known heterogeneity in genetic expression that can occur in gliomas, resulting in diverse tumor biology [17,48]. In clinical practice, immunohistochemistry (IHC) evaluations are mainly used to detect (using monoclonal antibodies) prevalent IDH mutations such as IDH1-R132H. Different cutoff values have been proposed for determining the IDH status using IHC methods, and heterogeneity in staining with IHC has been reported. While some advocate staining of more than 10% of tumor cells to confer IDH positivity, others suggest that one strongly stained tumor cell is sufficient [49]. Heterogeneity of staining with IHC has been reported, where up to 46% of subjects showed partial uptake [50]. In 2011, Perusser et al. reported that IDH1-R132H expression may occur in only a fraction of tumor cells [51]. Heterogeneity of the sample can also affect the sensitivity of genetic testing [52]. IDH heterogeneity and reported false negativity in some gliomas have been explained by monoallelic gene expression, wherein only one allele of a gene is expressed even though both alleles are present. According to Horbinski, sequencing may not always be adequate to identify tumors that are functionally IDH1/2 mutated [51,53]. Although heterogeneity of IDH status has been reported in histochemical and genomic evaluations of gliomas, we do not claim that the deep learning networks in this study are able to detect such heterogeneity in IDH mutation status. Rather, the mixed classification results likely reflect the morphologic heterogeneity of the IDH mutation status within a given tumor. Regardless, the accuracies using this voxel-wise approach mean that it well outperforms other methods.

Although IHC methods are routinely used in the clinic, several exome sequencing studies have demonstrated that traditional IDH1 antibody testing using IHC methods fails to detect up to 15% of IDH-mutated gliomas [25,26]. There are several molecular methods that can be used to determine IDH mutation status from tissue. The current gold standard is the whole-genome Sanger DNA sequencing method. Next-generation sequencing (NGS) methods such as whole-exome sequencing (WES) and NGS cancer gene panels can also be used to determine IDH mutation status. However, these molecular methods can be limited by the amount of time, cost, and volume of tumor tissue required for testing [54].

The study employed minimal data pre-processing. As for the T2-only network, high-quality T2-weighted images are commonly obtained during clinical brain tumor diagnostic evaluations and are generally resistant to patient motion effects, allowing for acquisition within two minutes. On modern MRI scanners, high-quality T2w images can even be obtained in the presence of patient motion [55]. As such, the ability to solely use T2w images presents a significant advantage for clinical translation. This method was inspired by similar approaches used for identifying other genetic statuses [56].

Our pre-processing steps preserve the original image information without needing any region of interest or pre-segmentation of the tumor. Prior deep learning algorithms for MRI-based IDH classification often relied on explicit tumor pre-segmentation, whether manual or through a separate deep learning network [17,38]. These pre-segmentation steps add complexity and, in the case of manual procedures, hinder the development of a robust automated clinical workflow. Our network uniquely carries out simultaneous tumor segmentation, a natural output of the voxel-wise classification process.

In reviewing the performance of the various external datasets, we noticed that the UTSW dataset tended to provide less accuracy for the MC-net than the other held-out datasets. Approximately half of the UTSW data used in this study represent MR data acquired at other nearby regional imaging centers, from where patients were referred to UTSW for surgical management with their outside imaging data sent into our PACs. The remainder of the UTSW subject imaging data were acquired on our own scanners using our standardized clinical brain tumor protocols (across a variety of vendors and platforms). When separating the UTSW data into external imaging centers and internal UTSW imaging data, there was a large difference in IDH classification accuracy, with the internal UTSW data providing 95.3% accuracy and the external referral data providing only 87.7% accuracy. This represents a potentially interesting area of additional inquiry in terms of the performance of molecular classification algorithms related to image acquisition parameters, image quality, field strength, vendor, and other factors that may be important when considering clinical implementation.

## 5. Future Work

Deep learning models usually require substantial amounts of data to train effectively. Training on a large dataset allows the networks to learn the underlying patterns and relationships in the data, improving their ability to generalize to unseen examples. The training subjects used in this study were only from the TCIA and EGD databases. While the TCIA dataset is relatively small (227 subjects) compared to the sample sizes typically required for deep learning, when combined with the EGD dataset, it provided nearly 700 subjects for training. Despite this caveat, the data are representative of real-world clinical experience, with multi-parametric MR images from multiple institutions with diverse acquisition parameters and imaging vendor platforms. Additionally, the evaluations were performed on over 1100 subjects with true held-out and geographically diverse datasets.

As seen in our study, the performance of both T2-net and MC-net improved when trained on the combined dataset of TCIA + EGD compared to TCIA alone. This indicates that adding more training data can help to improve the performance of the algorithms. A larger and more diverse training dataset can lead to better generalization and robustness in the model, allowing it to perform well on various external datasets. This is particularly important for deep learning algorithms, as they tend to require large amounts of data to learn the underlying patterns and features necessary for accurate classification. Although our results show promise for clinical translation, our algorithms’ performance needs to be replicated on additional independent datasets with variable acquisition parameters and imaging vendor platforms.

## 6. Conclusions

We developed two MRI-based deep learning networks for IDH classification of gliomas, a T2w-image-only network (T2-net) and a multi-contrast network (MC-net), with high accuracy. The developed networks were tested on over 1100 true held-out subjects from diverse databases, making this the largest study on non-invasive, image-based IDH prediction to date. The MC-net outperformed the T2-net in terms of accuracy, sensitivity, specificity, AUC, and dice coefficient. However, T2-net has the advantage of using only T2w MR images, which are more widely available and have faster acquisition times. This makes the T2-net a promising tool for clinical settings where multi-contrast MR images may not be readily available. Our results support the potential integration of T2-net and MC-net in clinical practice to provide non-invasive, accurate, and rapid IDH mutation prediction.

## 7. Importance of the Study

The identification of isocitrate dehydrogenase (IDH) mutation status as a marker for treatment and prognosis is one of the most significant recent findings in brain glioma biology. Gliomas with the mutated form of the gene have a more favorable prognosis and respond better to treatment than those with the non-mutated or wildtype form. Currently, the only reliable method to establish IDH mutation status requires obtaining glioma tissue through an invasive brain biopsy or open surgical resection. The ability to determine IDH status non-invasively carries substantial importance in determining therapy and predicting prognosis. Two MRI-based deep learning networks were developed for IDH classification, a T2w-image-only network (T2-net) and a multi-contrast network (MC-net). The high IDH classification accuracy of our T2w-image-only network (T2-net) marks an important milestone in the journey towards clinical translation.

## Figures and Tables

**Table 1:** IDH status of all testing datasets included in this study.

	UTSW	NYU	UWM	EGD	UCSF	Total
**Mutated**	104	23	19	150	103	399
**Wildtype**	256	113	156	306	392	1223
**Total**	360	136	175	456	495	1622

**Table 2:** T2-net IDH classification results.

Network Type	Training Group	Metrics	UTSW	NYU	UWM	EGD	UCSF	Overall Accuracy	Overall AUC
**T2-net**	**TCIA**	Accuracy	83.9	77.9	85.1	85.3	88.7	85.4	0.8686
		Sensitivity	75.0	60.9	78.9	76.7	76.7	75.4	
		Specificity	87.5	81.4	85.9	89.5	91.8	88.6	
		Dice Score	0.83 ± 0.18	0.87 ± 0.14	0.84 ± 0.16	0.73 ± 0.19	0.82 ± 0.16	0.80 ± 0.18	
	**TCIA + EGD**	Accuracy	86.1	81.6	89.7	-	89.5	87.6	0.8931
		Sensitivity	81.7	60.9	84.2	-	70.9	75.5	
		Specificity	87.9	85.8	90.4	-	94.4	90.8	
		Dice Score	0.85 ± 0.15	0.88 ± 0.13	0.85 ± 0.15	-	0.83 ± 0.15	0.85 ± 0.15	

**Table 3:** MC-net IDH classification results.

Network Type	Training Group	Metrics	UTSW	NYU	UWM	EGD	UCSF	Overall Accuracy	Overall AUC
**MC-net**	**TCIA**	Accuracy	87.2	91.9	87.4	92.3	93.5	91.0	0.9448
		Sensitivity	73.1	78.3	84.2	86.0	89.3	83.0	
		Specificity	93.0	94.7	87.8	95.4	94.5	93.6	
		Dice Score	0.90 ± 0.13	0.92 ± 0.10	0.91 ± 0.11	0.77 ± 0.17	0.87 ± 0.14	0.86 ± 0.15	
	**TCIA + EGD**	Accuracy	90.0	92.6	92.6	-	94.9	92.8	0.9646
		Sensitivity	80.8	79.3	68.4	-	88.3	82.3	
		Specificity	93.8	96.5	95.5	-	96.7	95.6	
		Dice Score	0.90 ± 0.13	0.92 ± 0.13	0.92 ± 0.07	-	0.87 ± 0.13	0.89 ± 0.13	

**Table 4:** Mean voxel-wise accuracies for each network.

Network Type	Training Group	IDH Type	UTSW	NYU	UWM	EGD	UCSF	Overall
**T2-net**	TCIA	Mutant	71.05	56.95	76.40	74.97	73.49	72.60
		Wildtype	84.05	80.59	85.20	87.07	88.71	86.13
	TCIA + EGD	Mutant	78.48	62.10	81.94	-	70.18	73.79
		Wildtype	84.63	83.03	87.65	-	91.99	88.09
**MC-net**	TCIA	Mutant	71.17	69.43	81.11	81.57	84.92	79.00
		Wildtype	90.57	91.37	85.26	93.11	91.18	90.80
	TCIA + EGD	Mutant	80.01	73.64	69.65	-	85.68	80.98
		Wildtype	91.71	94.15	93.33	-	93.50	93.05

## Data Availability

The results <published or shown> here are in whole or part based upon data generated by the TCGA Research Network: http://cancergenome.nih.gov/: TCGA-GBM (DOI: 10.7937/K9/TCIA.2016.RNYFUYE9) , TCGA-LGG (DOI: 10.7937/K9/TCIA.2016.L4LTD3TK) and Ivy GAP (DOI: 10.7937/K9/TCIA.2016.XLwaN6nL). Results were also derived from UCSF-PDGM (DOI: 10.7937/tcia.bdgf-8v37), and EGD (https://xnat.bmia.nl/data/archive/projects/egd) publicly available datasets. Data supporting the findings of this study that are not already included in the article or supplementary materials may be available from the authors upon reasonable request. Some data may be restricted due to privacy and/or ethical restrictions.

## Supplementary Materials

The following supporting information can be downloaded at: https://www.mdpi.com/article/10.3390/bioengineering10091045/s1; Table S1. Training subjects and corresponding IDH status; Table S2. Hyperparameters used to develop the MC-net.
