# Supplementary material for: MRI-Based Deep Learning Method for Classification of IDH Mutation Status
Source: Bioengineering (Basel). 2023 Sep 5;10(9):1045. doi: 10.3390/bioengineering10091045 (PMC10525372; doi:10.3390/bioengineering10091045)
Supplement: Supplementary file 1 [file bioengineering-10-01045-s001.zip › bioengineering-2564264-supplementary.pdf]

bioengineering

MDPI

Supplementary Materials

MRI-Based Deep Learning Method for Classification of IDH Mutation Status

Chandan Ganesh Bangalore Yogananda [1,*], Benjamin C. Wagner [1], Nghi C. D. Truong [1], James M. Holcomb [1], Divya D. Reddy [1], Niloufar Saadat [1], Kimmo J. Hatanpaa [2], Toral R. Patel [3], Baowei Fei [1,4], Matthew D. Lee [5], Rajan Jain [5,6], Richard J. Bruce [7], Marco C. Pinho [1], Ananth J. Madhuranthakam [1] and Joseph A. Maldjian [1]

1 Department of Radiology, University of Texas Southwestern Medical Center, 75390 Dallas, TX, USA; ben.wagner@utsouthwestern.edu (B.C.W.); nghi.truong@utsouthwestern.edu (N.C.D.T.); james.holcomb@utsouthwestern.edu (J.M.H.); divya.reddy@utsouthwestern.edu (D.D.R.); niloufar.saadat@utsouthwestern.edu (N.S.); bfei@utdallas.edu (B.F.); marco.pinho@utsouthwestern.edu (M.C.P.); ananth.madhuranthakam@utsouthwestern.edu (A.J.M.); joseph.maldjian@utsouthwestern.edu (J.A.M.)
2 Department of Pathology, University of Texas Southwestern Medical Center, 75390 Dallas, TX, USA; kimmo.hatanpaa@utsouthwestern.edu
3 Department of Neurological Surgery, University of Texas Southwestern Medical Center, 75390 Dallas, TX, USA; toral.patel@utsouthwestern.edu
4 Department of Bioengineering, University of Texas at Dallas, 75080 Richardson, TX, USA
5 Department of Radiology, NYU Grossman School of Medicine, 10016 New York, NY, USA; matthew.lee4@nyulangone.org (M.D.L.); rajan.jain@nyulangone.org (R.J.)
6 Department of Neurosurgery, NYU Grossman School of Medicine, 10016 New York, NY, USA
7 Department of Radiology, University of Wisconsin School of Medicine and Public Health, 53726 Madison, WI, USA; rbruce@uwhealth.org
* Correspondence: chandanganesh.bangaloreyogananda@utsouthwestern.edu

1. ROC Methodology

The network output classifies voxels in the tumor as IDH mutated or IDH wildtype. The percentage of IDH-mutated voxels was computed for the network output for each subject in the test set by dividing the predicted IDH-mutated voxels by the total number of predicted voxels in each tumor. The percentage of mutated voxels can be viewed as a network output prediction likelihood of the tumor being IDH mutated. Note that, in the manuscript, majority voting (the 50% threshold) was used to determine IDH prediction. For the ROC analysis, the percentage of IDH-mutated voxels was sorted and used as separate thresholds (cut points) to determine IDH mutation status for the subjects across the test set for each new cut point. The resulting predicted IDH class membership was compared to the ground truth values to determine sensitivity (true-positive rate) and 1- specificity (false-positive rate) at each threshold. The resulting values were plotted using R programming to obtain an ROC curve (true-positive rate against false-positive rate). Routines in R programming were used to fit the curves and determine the area under the curve (AUC). This procedure was repeated for both T2-net and MC-net and their two training combinations. Smoothed curves were used to facilitate displaying the figure.

Table S1. Training subjects and corresponding IDH status.

Subject ID	IDH Status
TCGA-06-0128_19990218	mutated
TCGA-06-0129_20000314	mutated
TCGA-06-2570_20070726	mutated
TCGA-06-5417_20080903	mutated
TCGA-06-6389_20090404	mutated
TCGA-14-1456_19990422	mutated
TCGA-CS-4942_19970222	mutated
TCGA-CS-4943_20000902	mutated
TCGA-CS-4944_20010208	mutated
TCGA-CS-5393_19990606	mutated
TCGA-CS-5396_20010302	mutated
TCGA-CS-6290_20000917	mutated
TCGA-CS-6665_20010817	mutated
TCGA-CS-6666_20011109	mutated
TCGA-CS-6667_20011105	mutated
TCGA-CS-6668_20011025	mutated
TCGA-DU-5849_19950405	mutated
TCGA-DU-5851_19950428	mutated
TCGA-DU-5853_19950823	mutated
TCGA-DU-5855_19951217	mutated
TCGA-DU-5871_19941206	mutated
TCGA-DU-5872_19950223	mutated
TCGA-DU-5874_19950510	mutated
TCGA-DU-6395_19830209	mutated
TCGA-DU-6397_19850130	mutated
TCGA-DU-6399_19830416	mutated
TCGA-DU-6400_19830518	mutated
TCGA-DU-6401_19831101	mutated
TCGA-DU-6407_19860514	mutated
TCGA-DU-6408_19860521	mutated
TCGA-DU-7008_19830723	mutated
TCGA-DU-7010_19860307	mutated
TCGA-DU-7015_19890618	mutated
TCGA-DU-7018_19911220	mutated
TCGA-DU-7019_19940908	mutated
TCGA-DU-7294_19890104	mutated
TCGA-DU-7298_19910324	mutated
TCGA-DU-7299_19910417	mutated
TCGA-DU-7300_19910814	mutated
TCGA-DU-7301_19911112	mutated
TCGA-DU-7302_19911203	mutated
TCGA-DU-7304_19930325	mutated
TCGA-DU-7306_19930512	mutated
TCGA-DU-7309_19960831	mutated
TCGA-DU-8164_19970111	mutated
TCGA-DU-8166_19970322	mutated
TCGA-DU-8167_19970402	mutated
TCGA-DU-8168_19970503	mutated
TCGA-DU-A5TP_19970614	mutated
TCGA-DU-A5TR_19970726	mutated
TCGA-DU-A5TS_19970726	mutated
TCGA-DU-A5TU_19980312	mutated
TCGA-DU-A5TW_19980228	mutated
TCGA-DU-A6S2_19980404	mutated
TCGA-DU-A6S3_19980711	mutated
TCGA-DU-A6S6_19920521	mutated
TCGA-DU-A6S7_19980513	mutated
TCGA-DU-A6S8_19980620	mutated
TCGA-FG-5964_20010511	mutated
TCGA-FG-6689_20020326	mutated
TCGA-FG-6690_20020226	mutated
TCGA-FG-6691_20020405	mutated
TCGA-FG-7634_20000128	mutated
TCGA-FG-8189_20030516	mutated
TCGA-FG-A4MT_20020212	mutated
TCGA-FG-A6IZ_20040220	mutated
TCGA-FG-A713_20040709	mutated
TCGA-HT-7473_19970826	mutated
TCGA-HT-7475_19970918	mutated
TCGA-HT-7602_19951103	mutated
TCGA-HT-7605_19950916	mutated
TCGA-HT-7608_19940304	mutated
TCGA-HT-7616_19940813	mutated
TCGA-HT-7684_19950816	mutated
TCGA-HT-7686_19950629	mutated
TCGA-HT-7690_19960312	mutated
TCGA-HT-7692_19960724	mutated
TCGA-HT-7693_19950520	mutated
TCGA-HT-7694_19950404	mutated
TCGA-HT-7855_19951020	mutated
TCGA-HT-7856_19950831	mutated
TCGA-HT-7874_19950902	mutated
TCGA-HT-7879_19981009	mutated
TCGA-HT-7884_19980913	mutated
TCGA-HT-8018_19970411	mutated
TCGA-HT-8105_19980826	mutated
TCGA-HT-8106_19970727	mutated
TCGA-HT-8111_19980330	mutated
TCGA-HT-8113_19930809	mutated
TCGA-HT-8114_19981030	mutated
TCGA-HT-8563_19981209	mutated
TCGA-HT-A61A_20000127	mutated
W153	mutated
W21	mutated
TCGA-02-0003_19970608	wildtype
TCGA-02-0006_19960823	wildtype
TCGA-02-0009_19970614	wildtype
TCGA-02-0011_19980201	wildtype
TCGA-02-0027_19990328	wildtype
TCGA-02-0033_19970526	wildtype
TCGA-02-0034_19970727	wildtype
TCGA-02-0037_19980113	wildtype
TCGA-02-0046_19981128	wildtype
TCGA-02-0047_19981215	wildtype
TCGA-02-0048_19990129	wildtype
TCGA-02-0060_20000228	wildtype
TCGA-02-0064_19990808	wildtype
TCGA-02-0068_20000516	wildtype
TCGA-02-0069_20000528	wildtype
TCGA-02-0070_20000710	wildtype
TCGA-02-0075_19990924	wildtype
TCGA-02-0086_19990725	wildtype
TCGA-02-0102_19971215	wildtype
TCGA-06-0119_20031226	wildtype
TCGA-06-0122_20040914	wildtype
TCGA-06-0132_20050517	wildtype
TCGA-06-0133_20050510	wildtype
TCGA-06-0137_20011224	wildtype
TCGA-06-0138_20021125	wildtype
TCGA-06-0142_20000311	wildtype
TCGA-06-0143_20050211	wildtype
TCGA-06-0145_20001106	wildtype
TCGA-06-0147_19970610	wildtype
TCGA-06-0154_19960405	wildtype
TCGA-06-0158_19960905	wildtype
TCGA-06-0166_19990601	wildtype
TCGA-06-0168_20000812	wildtype
TCGA-06-0173_20011105	wildtype
TCGA-06-0174_20011109	wildtype
TCGA-06-0176_20020409	wildtype

TCGA-06-0184_20030713	wildtype
TCGA-06-0185_20031107	wildtype
TCGA-06-0187_20040707	wildtype
TCGA-06-0188_20040717	wildtype
TCGA-06-0189_20041002	wildtype
TCGA-06-0190_20041210_DUP	wildtype
TCGA-06-0192_20050408	wildtype
TCGA-06-0213_19961023	wildtype
TCGA-06-0237_20050201	wildtype
TCGA-06-0238_20050412	wildtype
TCGA-06-0241_20050830	wildtype
TCGA-06-0644_20051128	wildtype
TCGA-06-0645_20051130	wildtype
TCGA-06-0646_20051209	wildtype
TCGA-06-0648_20060120	wildtype
TCGA-06-0649_20060126	wildtype
TCGA-06-0881_20070208	wildtype
TCGA-06-1806_20070602	wildtype
TCGA-06-5412_20080603	wildtype
TCGA-06-5413_20080617	wildtype
TCGA-12-0616_19990412	wildtype
TCGA-12-0829_19990602	wildtype
TCGA-12-1093_19990920	wildtype
TCGA-12-1598_19990823	wildtype
TCGA-12-1601_20000619	wildtype
TCGA-12-1602_20010304	wildtype
TCGA-12-3650_20010729	wildtype
TCGA-14-0789_19971119	wildtype
TCGA-14-1794_19980411	wildtype
TCGA-14-1829_20010614	wildtype
TCGA-14-3477_20020501	wildtype
TCGA-19-1390_20010320	wildtype
TCGA-19-1789_20020810	wildtype
TCGA-19-2624_20021210	wildtype
TCGA-19-2631_20030302	wildtype
TCGA-19-5954_20031230	wildtype
TCGA-19-5958_20040309	wildtype
TCGA-27-1835_19880731	wildtype
TCGA-27-1838_19880829	wildtype
TCGA-76-4926_19960329	wildtype
TCGA-76-4932_19970316	wildtype
TCGA-76-4934_20001008	wildtype
TCGA-76-4935_20010122	wildtype
TCGA-76-6191_20001020	wildtype
TCGA-76-6192_20010525	wildtype
TCGA-76-6193_20010602	wildtype
TCGA-76-6280_19980721	wildtype
TCGA-76-6282_19980824	wildtype
TCGA-76-6285_19981219	wildtype
TCGA-76-6656_20010602	wildtype
TCGA-76-6657_20010611	wildtype
TCGA-76-6661_20011208	wildtype
TCGA-76-6662_20011213	wildtype
TCGA-76-6663_20011227	wildtype
TCGA-76-6664_20020110	wildtype
TCGA-CS-4941_19960909	wildtype
TCGA-CS-5395_19981004	wildtype
TCGA-CS-5397_20010315	wildtype
TCGA-CS-6186_20000601	wildtype
TCGA-CS-6188_20010812	wildtype
TCGA-CS-6669_20020102	wildtype
TCGA-DU-5852_19950709	wildtype
TCGA-DU-5854_19951104	wildtype
TCGA-DU-6404_19850629	wildtype
TCGA-DU-6405_19851005	wildtype
TCGA-DU-8162_19961029	wildtype
TCGA-DU-8165_19970205	wildtype
TCGA-DU-A5TT_19980318	wildtype
TCGA-DU-A5TY_19970709	wildtype
TCGA-FG-6688_20020215	wildtype
TCGA-FG-6692_20020606	wildtype
TCGA-HT-7680_19970202	wildtype
TCGA-HT-7860_19960513	wildtype
TCGA-HT-7882_19970125	wildtype
TCGA-HT-8107_19980708	wildtype
TCGA-HT-A5RC_19990831	wildtype
W100	wildtype
W102	wildtype
W187	wildtype
W2	wildtype
W23	wildtype
W26	wildtype
W3	wildtype
W5	wildtype
W57	wildtype
W6	wildtype
W7	wildtype
W71	wildtype
W74	wildtype
W76	wildtype
W80	wildtype
W84	wildtype
W9	wildtype
W91	wildtype
W93	wildtype
W96	wildtype
W98	wildtype
MR_EGD-0004	mutated
MR_EGD-0008	mutated
MR_EGD-0009	wildtype
MR_EGD-0011	mutated
MR_EGD-0014	wildtype
MR_EGD-0015	wildtype
MR_EGD-0020	mutated
MR_EGD-0022	mutated
MR_EGD-0024	mutated
MR_EGD-0026	wildtype
MR_EGD-0029	mutated
MR_EGD-0031	wildtype
MR_EGD-0033	mutated
MR_EGD-0034	wildtype
MR_EGD-0035	wildtype
MR_EGD-0041	wildtype
MR_EGD-0045	wildtype
MR_EGD-0047	mutated
MR_EGD-0050	wildtype
MR_EGD-0052	wildtype
MR_EGD-0053	wildtype
MR_EGD-0055	wildtype
MR_EGD-0057	mutated
MR_EGD-0058	wildtype
MR_EGD-0059	wildtype
MR_EGD-0062	mutated
MR_EGD-0064	wildtype
MR_EGD-0066	mutated
MR_EGD-0067	wildtype
MR_EGD-0068	mutated
MR_EGD-0072	mutated
MR_EGD-0073	wildtype
MR_EGD-0074	mutated
MR_EGD-0075	wildtype
MR_EGD-0078	mutated
MR_EGD-0080	wildtype
MR_EGD-0081	wildtype
MR_EGD-0083	wildtype
MR_EGD-0085	wildtype
MR_EGD-0086	mutated
MR_EGD-0088	wildtype

MR_EGD-0089	wildtype		MR_EGD-0171	wildtype		MR_EGD-0238	wildtype
MR_EGD-0090	mutated		MR_EGD-0172	wildtype		MR_EGD-0240	mutated
MR_EGD-0094	wildtype		MR_EGD-0176	wildtype		MR_EGD-0241	mutated
MR_EGD-0097	wildtype		MR_EGD-0177	mutated		MR_EGD-0242	wildtype
MR_EGD-0099	wildtype		MR_EGD-0179	mutated		MR_EGD-0243	mutated
MR_EGD-0100	wildtype		MR_EGD-0182	wildtype		MR_EGD-0244	mutated
MR_EGD-0101	mutated		MR_EGD-0183	wildtype		MR_EGD-0247	wildtype
MR_EGD-0102	wildtype		MR_EGD-0185	mutated		MR_EGD-0249	wildtype
MR_EGD-0105	wildtype		MR_EGD-0186	mutated		MR_EGD-0250	wildtype
MR_EGD-0106	mutated		MR_EGD-0187	mutated		MR_EGD-0253	mutated
MR_EGD-0107	mutated		MR_EGD-0188	mutated		MR_EGD-0254	wildtype
MR_EGD-0109	wildtype		MR_EGD-0191	mutated		MR_EGD-0255	wildtype
MR_EGD-0110	wildtype		MR_EGD-0192	wildtype		MR_EGD-0256	wildtype
MR_EGD-0111	wildtype		MR_EGD-0193	wildtype		MR_EGD-0262	wildtype
MR_EGD-0112	wildtype		MR_EGD-0194	mutated		MR_EGD-0263	wildtype
MR_EGD-0114	wildtype		MR_EGD-0196	wildtype		MR_EGD-0264	wildtype
MR_EGD-0115	wildtype		MR_EGD-0197	wildtype		MR_EGD-0266	wildtype
MR_EGD-0117	wildtype		MR_EGD-0198	wildtype		MR_EGD-0268	mutated
MR_EGD-0118	mutated		MR_EGD-0199	mutated		MR_EGD-0270	wildtype
MR_EGD-0121	wildtype		MR_EGD-0202	wildtype		MR_EGD-0273	wildtype
MR_EGD-0122	mutated		MR_EGD-0203	mutated		MR_EGD-0275	wildtype
MR_EGD-0123	mutated		MR_EGD-0204	wildtype		MR_EGD-0276	mutated
MR_EGD-0126	wildtype		MR_EGD-0206	wildtype		MR_EGD-0279	mutated
MR_EGD-0127	wildtype		MR_EGD-0208	mutated		MR_EGD-0280	wildtype
MR_EGD-0128	mutated		MR_EGD-0211	mutated		MR_EGD-0281	wildtype
MR_EGD-0131	wildtype		MR_EGD-0213	wildtype		MR_EGD-0282	wildtype
MR_EGD-0132	wildtype		MR_EGD-0214	mutated		MR_EGD-0285	wildtype
MR_EGD-0133	mutated		MR_EGD-0215	wildtype		MR_EGD-0286	mutated
MR_EGD-0134	wildtype		MR_EGD-0216	wildtype		MR_EGD-0290	wildtype
MR_EGD-0137	mutated		MR_EGD-0217	mutated		MR_EGD-0292	wildtype
MR_EGD-0141	wildtype		MR_EGD-0218	wildtype		MR_EGD-0293	wildtype
MR_EGD-0143	mutated		MR_EGD-0219	wildtype		MR_EGD-0294	wildtype
MR_EGD-0144	wildtype		MR_EGD-0221	mutated		MR_EGD-0297	mutated
MR_EGD-0145	mutated		MR_EGD-0222	wildtype		MR_EGD-0298	wildtype
MR_EGD-0146	mutated		MR_EGD-0223	wildtype		MR_EGD-0299	wildtype
MR_EGD-0152	wildtype		MR_EGD-0224	wildtype		MR_EGD-0301	mutated
MR_EGD-0155	wildtype		MR_EGD-0226	wildtype		MR_EGD-0302	wildtype
MR_EGD-0156	wildtype		MR_EGD-0227	mutated		MR_EGD-0303	wildtype
MR_EGD-0158	wildtype		MR_EGD-0228	mutated		MR_EGD-0304	wildtype
MR_EGD-0160	mutated		MR_EGD-0229	mutated		MR_EGD-0305	wildtype
MR_EGD-0161	wildtype		MR_EGD-0230	mutated		MR_EGD-0306	mutated
MR_EGD-0162	mutated		MR_EGD-0232	wildtype		MR_EGD-0308	mutated
MR_EGD-0165	wildtype		MR_EGD-0233	mutated		MR_EGD-0311	wildtype
MR_EGD-0166	wildtype		MR_EGD-0234	wildtype		MR_EGD-0312	mutated
MR_EGD-0167	wildtype		MR_EGD-0236	mutated		MR_EGD-0313	wildtype
MR_EGD-0168	wildtype		MR_EGD-0237	wildtype		MR_EGD-0314	wildtype

MR_EGD-0315	wildtype
MR_EGD-0317	wildtype
MR_EGD-0318	wildtype
MR_EGD-0319	wildtype
MR_EGD-0321	wildtype
MR_EGD-0323	wildtype
MR_EGD-0324	wildtype
MR_EGD-0325	wildtype
MR_EGD-0328	wildtype
MR_EGD-0329	wildtype
MR_EGD-0330	mutated
MR_EGD-0331	wildtype
MR_EGD-0332	mutated
MR_EGD-0334	wildtype
MR_EGD-0336	wildtype
MR_EGD-0337	wildtype
MR_EGD-0338	wildtype
MR_EGD-0340	mutated
MR_EGD-0342	wildtype
MR_EGD-0343	wildtype
MR_EGD-0344	wildtype
MR_EGD-0345	wildtype
MR_EGD-0346	mutated
MR_EGD-0349	wildtype
MR_EGD-0351	mutated
MR_EGD-0352	mutated
MR_EGD-0353	wildtype
MR_EGD-0354	wildtype
MR_EGD-0355	mutated
MR_EGD-0356	mutated
MR_EGD-0357	wildtype
MR_EGD-0358	wildtype
MR_EGD-0363	wildtype
MR_EGD-0365	wildtype
MR_EGD-0366	wildtype
MR_EGD-0367	wildtype
MR_EGD-0368	mutated
MR_EGD-0369	mutated
MR_EGD-0370	wildtype
MR_EGD-0375	wildtype
MR_EGD-0376	wildtype
MR_EGD-0377	mutated
MR_EGD-0378	mutated
MR_EGD-0380	wildtype
MR_EGD-0381	wildtype
MR_EGD-0382	wildtype

MR_EGD-0383	wildtype
MR_EGD-0388	wildtype
MR_EGD-0389	wildtype
MR_EGD-0390	wildtype
MR_EGD-0391	wildtype
MR_EGD-0392	wildtype
MR_EGD-0394	wildtype
MR_EGD-0395	wildtype
MR_EGD-0397	wildtype
MR_EGD-0400	wildtype
MR_EGD-0401	wildtype
MR_EGD-0402	mutated
MR_EGD-0403	mutated
MR_EGD-0405	wildtype
MR_EGD-0407	wildtype
MR_EGD-0410	mutated
MR_EGD-0411	mutated
MR_EGD-0412	wildtype
MR_EGD-0413	mutated
MR_EGD-0414	wildtype
MR_EGD-0415	mutated
MR_EGD-0416	wildtype
MR_EGD-0417	wildtype
MR_EGD-0418	wildtype
MR_EGD-0420	mutated
MR_EGD-0421	mutated
MR_EGD-0423	wildtype
MR_EGD-0424	wildtype
MR_EGD-0425	wildtype
MR_EGD-0426	mutated
MR_EGD-0427	wildtype
MR_EGD-0428	wildtype
MR_EGD-0430	wildtype
MR_EGD-0431	wildtype
MR_EGD-0432	wildtype
MR_EGD-0433	wildtype
MR_EGD-0434	mutated
MR_EGD-0438	wildtype
MR_EGD-0439	mutated
MR_EGD-0440	wildtype
MR_EGD-0442	wildtype
MR_EGD-0443	wildtype
MR_EGD-0447	wildtype
MR_EGD-0448	wildtype
MR_EGD-0449	wildtype
MR_EGD-0450	mutated

MR_EGD-0451	wildtype
MR_EGD-0452	wildtype
MR_EGD-0453	mutated
MR_EGD-0455	wildtype
MR_EGD-0460	wildtype
MR_EGD-0461	mutated
MR_EGD-0463	wildtype
MR_EGD-0464	wildtype
MR_EGD-0466	mutated
MR_EGD-0469	wildtype
MR_EGD-0470	wildtype
MR_EGD-0472	mutated
MR_EGD-0474	mutated
MR_EGD-0478	wildtype
MR_EGD-0479	wildtype
MR_EGD-0480	wildtype
MR_EGD-0482	wildtype
MR_EGD-0484	wildtype
MR_EGD-0485	wildtype
MR_EGD-0486	wildtype
MR_EGD-0487	mutated
MR_EGD-0488	wildtype
MR_EGD-0490	mutated
MR_EGD-0492	mutated
MR_EGD-0494	wildtype
MR_EGD-0495	mutated
MR_EGD-0499	mutated
MR_EGD-0501	wildtype
MR_EGD-0502	wildtype
MR_EGD-0503	wildtype
MR_EGD-0505	wildtype
MR_EGD-0506	wildtype
MR_EGD-0507	wildtype
MR_EGD-0508	wildtype
MR_EGD-0509	mutated
MR_EGD-0511	mutated
MR_EGD-0512	wildtype
MR_EGD-0514	mutated
MR_EGD-0515	wildtype
MR_EGD-0516	wildtype
MR_EGD-0518	mutated
MR_EGD-0521	mutated
MR_EGD-0522	wildtype
MR_EGD-0523	mutated
MR_EGD-0524	wildtype
MR_EGD-0529	wildtype

MR_EGD-0530	mutated
MR_EGD-0531	wildtype
MR_EGD-0532	wildtype
MR_EGD-0535	wildtype
MR_EGD-0536	mutated
MR_EGD-0537	mutated
MR_EGD-0539	wildtype
MR_EGD-0541	mutated
MR_EGD-0543	mutated
MR_EGD-0544	wildtype
MR_EGD-0545	wildtype
MR_EGD-0547	wildtype
MR_EGD-0548	wildtype
MR_EGD-0549	wildtype
MR_EGD-0552	mutated
MR_EGD-0553	mutated
MR_EGD-0557	wildtype
MR_EGD-0558	wildtype
MR_EGD-0561	mutated
MR_EGD-0563	mutated
MR_EGD-0564	mutated
MR_EGD-0565	mutated
MR_EGD-0567	mutated
MR_EGD-0568	mutated
MR_EGD-0571	mutated
MR_EGD-0572	mutated
MR_EGD-0575	wildtype
MR_EGD-0577	mutated
MR_EGD-0578	wildtype
MR_EGD-0579	wildtype
MR_EGD-0580	wildtype
MR_EGD-0581	wildtype
MR_EGD-0585	mutated
MR_EGD-0586	mutated
MR_EGD-0587	wildtype
MR_EGD-0589	wildtype
MR_EGD-0591	wildtype
MR_EGD-0592	wildtype
MR_EGD-0593	wildtype
MR_EGD-0594	wildtype
MR_EGD-0595	wildtype
MR_EGD-0596	wildtype
MR_EGD-0598	mutated
MR_EGD-0599	wildtype
MR_EGD-0600	wildtype
MR_EGD-0601	mutated

MR_EGD-0602	wildtype
MR_EGD-0603	wildtype
MR_EGD-0604	wildtype
MR_EGD-0605	wildtype
MR_EGD-0606	mutated
MR_EGD-0608	wildtype
MR_EGD-0611	wildtype
MR_EGD-0612	wildtype
MR_EGD-0617	wildtype
MR_EGD-0621	wildtype
MR_EGD-0622	wildtype
MR_EGD-0624	wildtype
MR_EGD-0626	mutated
MR_EGD-0627	wildtype
MR_EGD-0629	wildtype
MR_EGD-0631	mutated
MR_EGD-0632	wildtype
MR_EGD-0633	mutated
MR_EGD-0635	wildtype
MR_EGD-0636	mutated
MR_EGD-0638	mutated
MR_EGD-0639	wildtype
MR_EGD-0640	wildtype
MR_EGD-0641	wildtype
MR_EGD-0643	wildtype
MR_EGD-0645	wildtype
MR_EGD-0647	mutated
MR_EGD-0648	wildtype
MR_EGD-0655	mutated
MR_EGD-0656	mutated
MR_EGD-0657	wildtype
MR_EGD-0658	wildtype
MR_EGD-0659	wildtype
MR_EGD-0661	wildtype
MR_EGD-0662	mutated
MR_EGD-0663	wildtype
MR_EGD-0664	wildtype
MR_EGD-0665	wildtype
MR_EGD-0667	wildtype
MR_EGD-0668	mutated
MR_EGD-0669	wildtype
MR_EGD-0671	wildtype
MR_EGD-0672	wildtype
MR_EGD-0675	wildtype
MR_EGD-0676	wildtype
MR_EGD-0677	wildtype

MR_EGD-0687	wildtype
MR_EGD-0688	mutated
MR_EGD-0689	mutated
MR_EGD-0691	wildtype
MR_EGD-0692	wildtype
MR_EGD-0693	wildtype
MR_EGD-0694	wildtype
MR_EGD-0697	wildtype
MR_EGD-0699	wildtype
MR_EGD-0700	mutated
MR_EGD-0702	wildtype
MR_EGD-0703	wildtype
MR_EGD-0705	mutated
MR_EGD-0706	wildtype
MR_EGD-0707	wildtype
MR_EGD-0709	wildtype
MR_EGD-0710	wildtype
MR_EGD-0715	wildtype
MR_EGD-0720	mutated
MR_EGD-0723	wildtype
MR_EGD-0725	wildtype
MR_EGD-0729	mutated
MR_EGD-0734	wildtype
MR_EGD-0735	wildtype
MR_EGD-0736	wildtype
MR_EGD-0737	mutated
MR_EGD-0738	wildtype
MR_EGD-0739	mutated
MR_EGD-0741	wildtype
MR_EGD-0742	wildtype
MR_EGD-0744	mutated
MR_EGD-0745	wildtype
MR_EGD-0747	wildtype
MR_EGD-0753	wildtype
MR_EGD-0754	wildtype
MR_EGD-0757	mutated
MR_EGD-0758	mutated
MR_EGD-0759	wildtype
MR_EGD-0760	wildtype
MR_EGD-0762	wildtype
MR_EGD-0763	wildtype
MR_EGD-0764	wildtype
MR_EGD-0765	wildtype
MR_EGD-0766	wildtype
MR_EGD-0768	wildtype
MR_EGD-0771	mutated

MR_EGD-0772	wildtype
MR_EGD-0773	mutated

Table S2. Hyperparameters used to develop the MC-net.

Sl No.	Training Hyperparameters
1	"_best_ema": "0.8365259113358365",
2	"batch_size": "100",
3	"configuration_manager": "{'data_identifier': 'nnUNetPlans_2d', 'preprocessor_name': 'DefaultPreprocessor', 'batch_size': 100, 'patch_size': [256, 224], 'median_image_size_in_voxels': [233.0, 197.0], 'spacing': [1.0, 1.0], 'normalization_schemes': ['ZScoreNormalization', 'ZScoreNormalization', 'ZScoreNormalization', 'ZScoreNormalization'], 'use_mask_for_norm': [False, False, False, False], 'UNet_class_name': 'PlainConvUNet', 'UNet_base_num_features': 32, 'n_conv_per_stage_encoder': [2, 2, 2, 2, 2, 2], 'n_conv_per_stage_decoder': [2, 2, 2, 2, 2], 'num_pool_per_axis': [5, 5], 'pool_op_kernel_sizes': [[1, 1], [2, 2], [2, 2], [2, 2], [2, 2], [2, 2]], 'conv_kernel_sizes': [[3, 3], [3, 3], [3, 3], [3, 3], [3, 3], [3, 3]], 'unet_max_num_features': 512, 'resampling_fn_data': 'resample_data_or_seg_to_shape', 'resampling_fn_seg': 'resample_data_or_seg_to_shape', 'resampling_fn_data_kwargs': {'is_seg': False, 'order': 3, 'order_z': 0, 'force_separate_z': None}, 'resampling_fn_seg_kwargs': {'is_seg': True, 'order': 1, 'order_z': 0, 'force_separate_z': None}, 'resampling_fn_probabilities': 'resample_data_or_seg_to_shape', 'resampling_fn_probabilities_kwargs': {'is_seg': False, 'order': 1, 'order_z': 0, 'force_separate_z': None}, 'batch_dice': True}",
4	"configuration_name": "2d",
5	"cudnn_version": 8500,
6	"current_epoch": "1250",
7	"dataloader_train": "<nnunetv2.training.data_augmentation.custom_transforms.limited_length_multithreaded_augmenter.LimitedLenWrapper object at 0x2aabf8d86020>",
8	"dataloader_train.generator": "<nnunetv2.training.dataloading.data_loader_2d.nnUNetDataLoader2D object at 0x2aabf8d86b60>",
9	"dataloader_train.num_processes": "100",
10	"dataloader_train.transform": "Compose ([SpatialTransform(independent_scale_for_each_axis = True, p_rot_per_sample = 0.4, p_scale_per_sample = 0.2, p_el_per_sample = 0.2, data_key = 'data', label_key = 'seg', patch_size = [256, 224], patch_center_dist_from_border = None, do_elastic_deform = False, alpha = (0.0, 1000.0), sigma = (10.0, 13.0), do_rotation = True, angle_x = (-3.141592653589793, 3.141592653589793), angle_y = (0, 0), angle_z = (0, 0), do_scale = True, scale = (0.7, 1.43), border_mode_data = 'constant', border_cval_data = 0, order_data = 3, border_mode_seg = 'constant', border_cval_seg = -1, order_seg = 1, random_crop = False, p_rot_per_axis = 0.5, p_independent_scale_per_axis = 1), OneOfTransform(list_of_transforms = [MedianFilterTransform(p_per_sample = 0.2, p_per_channel = 0.5, data_key = 'data', filter_size = (2, 8), same_for_each_channel = False), GaussianBlurTransform(p_per_sample = 0.2, different_sigma_per_channel = True, p_per_channel = 0.5, data_key = 'data', blur_sigma = (0.3, 1.5), different_sigma_per_axis = False, p_isotropic = 0)]), GaussianNoiseTransform(p_per_sample = 0.1, data_key = 'data', noise_variance = (0, 0.1), p_per_channel = 1, per_channel = False), BrightnessTransform(p_per_sample = 0.1, data_key = 'data', mu = 0, sigma = 0.5, per_channel = True, p_per_channel = 0.5), OneOfTransform(list_of_transforms = [ContrastAugmentationTransform(p_per_sample = 0.2, data_key = 'data', contrast_range = (0.5, 2),

	preserve_range = True, per_channel = True, p_per_channel = 0.5), ContrastAugmentationTransform(p_per_sample = 0.2, data_key = 'data', contrast_range = (0.5, 2), preserve_range = False, per_channel = True, p_per_channel = 0.5)]), SimulateLowResolutionTransform(order_upsample = 3, order_downsample = 0, channels = None, per_channel = True, p_per_channel = 0.5, p_per_sample = 0.15, data_key = 'data', zoom_range = (0.25, 1), ignore_axes = None), GammaTransform(p_per_sample = 0.1, retain_stats = True, per_channel = True, data_key = 'data', gamma_range = (0.7, 1.5), invert_image = True), GammaTransform(p_per_sample = 0.1, retain_stats = True, per_channel = True, data_key = 'data', gamma_range = (0.7, 1.5), invert_image = True), MirrorTransform(p_per_sample = 1, data_key = 'data', label_key = 'seg', axes = (0, 1)), BlankRectangleTransform(rectangle_size = [[25, 85], [22, 74]], num_rectangles = (1, 5), force_square = False, p_per_sample = 0.4, p_per_channel = 0.5, apply_to_keys = ('data',), color_fn = <function BlankRectangleTransform.__init__.<locals>.<lambda> at 0x2aabf4b8b2e0>), <batchgenerators.transforms.local_transforms.BrightnessGradientAdditiveTransform object at 0x2aabf8d85960>, <batchgenerators.transforms.local_transforms.LocalGammaTransform object at 0x2aabf8d85930>, SharpeningTransform(p_per_sample = 0.2, p_per_channel = 0.5, data_key = 'data', strength = (0.1, 1), same_for_each_channel = False), RemoveLabelTransform(output_key = 'seg', input_key = 'seg', replace_with = 0, remove_label = -1), RenameTransform(delete_old = True, out_key = 'target', in_key = 'seg'), DownsampleSegForDSTransform2(axes = None, output_key = 'target', input_key = 'target', order = 0, ds_scales = [[1.0, 1.0], [0.5, 0.5], [0.25, 0.25], [0.125, 0.125], [0.0625, 0.0625]]), NumpyToTensor(keys = ['data', 'target'], cast_to = 'float')])",
11	"dataloader_val": "<nnunetv2.training.data_augmentation.custom_transforms.limited_length_multithreaded_augmenter.LimitedLenWrapper object at 0x2aabf8d849d0>",
12	"dataloader_val.generator": "<nnunetv2.training.dataloading.data_loader_2d.nnUNetDataLoader2D object at 0x2aabf8d86da0>",
13	"dataloader_val.num_processes": "100",
14	"dataloader_val.transform": "Compose ([RemoveLabelTransform(output_key = 'seg', input_key = 'seg', replace_with = 0, remove_label = -1), RenameTransform(delete_old = True, out_key = 'target', in_key = 'seg'), DownsampleSegForDSTransform2(axes = None, output_key = 'target', input_key = 'target', order = 0, ds_scales = [[1.0, 1.0], [0.5, 0.5], [0.25, 0.25], [0.125, 0.125], [0.0625, 0.0625]]), NumpyToTensor(keys = ['data', 'target'], cast_to = 'float')])",
15	"dataset_json": "{'channel_names': {'T1': 0, 'T1C': 1, 'T2': 2, 'Flair': 3}, 'labels': {'background': 0, 'mutated': 1, 'wild-type': 2}, 'numTraining': 13660, 'file_ending': '.nii.gz', 'regions_class_order': 'TCIA FeTS002 and EGD MCon data --> SS Tio DA and with N4'}",
16	"device": "cuda:0",
17	"disable_checkpointing": "False",
18	"fold": "1",
19	"folder_with_segs_from_previous_stage": "None",
20	"gpu_name": "NVIDIA A100-PCIE-40GB",
21	"grad_scaler": "<torch.cuda.amp.grad_scaler.GradScaler object at 0x2aabf4b6d900>",
22	"hostname": "NucleusC092",
23	"inference_allowed_mirroring_axes": "(0, 1)",

24	"initial_lr": "0.01",
25	"is_cascaded": "False",
26	"is_ddp": "False",
27	"label_manager": "<nnunetv2.utilities.label_handling.label_handling.LabelManager object at 0x2aabf4b6d870>",
28	"local_rank": "0",
30	"logger": "<nnunetv2.training.logging.nnunet_logger.nnUNetLogger object at 0x2aabf4b6d7b0>",
31	"loss": "DeepSupervisionWrapper(\n (loss): DC_and_CE_loss(\n (ce): RobustCrossEntropyLoss()\n (dc): MemoryEfficientSoftDiceLoss()\n)\n)",
32	"lr_scheduler": "<nnunetv2.training.lr_scheduler.polylr.PolyLRScheduler object at 0x2aabf4b6e6e0>",
33	"my_init_kwargs": "{'plans': {'dataset_name': 'Dataset006_TCIA_FeTS002_EGD_SS_N4_BG_Tio_MCon_data', 'plans_name': 'nnUNetPlans', 'original_median_spacing_after_transp': [1.0, 1.0, 1.0], 'original_median_shape_after_transp': [189, 233, 197], 'image_reader_writer': 'SimpleITKIO', 'transpose_forward': [0, 1, 2], 'transpose_backward': [0, 1, 2], 'configurations': {'2d': {'data_identifier': 'nnUNetPlans_2d', 'preprocessor_name': 'DefaultPreprocessor', 'batch_size': 100, 'patch_size': [256, 224], 'median_image_size_in_voxels': [233.0, 197.0], 'spacing': [1.0, 1.0], 'normalization_schemes': ['ZScoreNormalization', 'ZScoreNormalization', 'ZScoreNormalization', 'ZScoreNormalization'], 'use_mask_for_norm': [False, False, False, False], 'UNet_class_name': 'PlainConvUNet', 'UNet_base_num_features': 32, 'n_conv_per_stage_encoder': [2, 2, 2, 2, 2, 2], 'n_conv_per_stage_decoder': [2, 2, 2, 2, 2], 'num_pool_per_axis': [5, 5], 'pool_op_kernel_sizes': [[1, 1], [2, 2], [2, 2], [2, 2], [2, 2], [2, 2]], 'conv_kernel_sizes': [[3, 3], [3, 3], [3, 3], [3, 3], [3, 3], [3, 3]], 'unet_max_num_features': 512, 'resampling_fn_data': 'resample_data_or_seg_to_shape', 'resampling_fn_seg': 'resample_data_or_seg_to_shape', 'resampling_fn_data_kwargs': {'is_seg': False, 'order': 3, 'order_z': 0, 'force_separate_z': None}, 'resampling_fn_seg_kwargs': {'is_seg': True, 'order': 1, 'order_z': 0, 'force_separate_z': None}, 'resampling_fn_probabilities': 'resample_data_or_seg_to_shape', 'resampling_fn_probabilities_kwargs': {'is_seg': False, 'order': 1, 'order_z': 0, 'force_separate_z': None}, 'batch_dice': True}, '3d_fullres': {'data_identifier': 'nnUNetPlans_3d_fullres', 'preprocessor_name': 'DefaultPreprocessor', 'batch_size': 2, 'patch_size': [192, 256, 192], 'median_image_size_in_voxels': [189.0, 233.0, 197.0], 'spacing': [1.0, 1.0, 1.0], 'normalization_schemes': ['ZScoreNormalization', 'ZScoreNormalization', 'ZScoreNormalization', 'ZScoreNormalization'], 'use_mask_for_norm': [False, False, False, False], 'UNet_class_name': 'PlainConvUNet', 'UNet_base_num_features': 32, 'n_conv_per_stage_encoder': [2, 2, 2, 2, 2, 2], 'n_conv_per_stage_decoder': [2, 2, 2, 2, 2], 'num_pool_per_axis': [5, 5, 5], 'pool_op_kernel_sizes': [[1, 1, 1], [2, 2, 2], [2, 2, 2], [2, 2, 2], [2, 2, 2], [2, 2, 2]], 'conv_kernel_sizes': [[3, 3, 3], [3, 3, 3], [3, 3, 3], [3, 3, 3], [3, 3, 3], [3, 3, 3]], 'unet_max_num_features': 320, 'resampling_fn_data': 'resample_data_or_seg_to_shape', 'resampling_fn_seg': 'resample_data_or_seg_to_shape', 'resampling_fn_data_kwargs': {'is_seg': False, 'order': 3, 'order_z': 0, 'force_separate_z': None}, 'resampling_fn_seg_kwargs': {'is_seg': True, 'order': 1, 'order_z': 0, 'force_separate_z': None}, 'resampling_fn_probabilities': 'resample_data_or_seg_to_shape', 'resampling_fn_probabilities_kwargs': {'is_seg': False, 'order': 1, 'order_z': 0, 'force_separate_z': None}, 'batch_dice': False}}, 'experiment_planner_used': 'ExperimentPlanner', 'label_manager': 'LabelManager', 'foreground_intensity_properties_per_channel': {'0': {'max': 8449.001953125, 'mean': 876.2181396484375, 'median': 587.5035400390625, 'min': -62.86849594116211, 'percentile_00_5': 0.0, 'percentile_99_5': 5023.051276855462, 'std': 860.8373413085938}, '1': {'max': 22296.71484375, 'mean': 1010.2324829101562,

	'median': 643.4981079101562, 'min': -217.8645782470703, 'percentile_00_5': 0.0, 'percentile_99_5': 6990.7314453125, 'std': 1197.094970703125}, '2': {'max': 13443.9501953125, 'mean': 906.3700561523438, 'median': 697.4663696289062, 'min': -311.94610595703125, 'percentile_00_5': 0.0, 'percentile_99_5': 4928.394047851558, 'std': 798.8444213867188}, '3': {'max': 11528.87890625, 'mean': 741.3021240234375, 'median': 524.4417724609375, 'min': -217.5197296142578, 'percentile_00_5': 0.0, 'percentile_99_5': 3948.712703857367, 'std': 668.06103515625}}}, 'configuration': '2d', 'fold': 1, 'dataset_json': {'channel_names': {'T1': 0, 'T1C': 1, 'T2': 2, 'Flair': 3}, 'labels': {'background': 0, 'mutated': 1, 'wild-type': 2}, 'numTraining': 13660, 'file_ending': '.nii.gz', 'regions_class_order': 'TCIA FeTS002 and EGD MCon data --> SS Tio DA and with N4'}, 'unpack_dataset': True, 'device': device(type='cuda')}",
34	"network": "PlainConvUNet",
35	"num_epochs": "2000",
36	"num_input_channels": "4",
37	"num_iterations_per_epoch": "250",
38	"num_val_iterations_per_epoch": "50",
39	"optimizer": "SGD (\nParameter Group 0\n dampening: 0\n differentiable: False\n foreach: None\n initial_lr: 0.01\n lr: 0.004141416859964053\n maximize: False\n momentum: 0.99\n nesterov: True\n weight_decay: 3e-05\n)",
41	"output_folder_base": "/project/radiology/ANSIR_lab/CLINICAL/UTSW/BrainTumor/shared/Chandan_processed_subjects/nnUNet_V2_database/nnUNet_trained_models/Dataset006_TCIA_FeTS002_EGD_SS_N4_BG_Tio_MCon_data/nnUNetTrainerDA5_2000epochs__nnUNetPlans__2d",
42	"oversample_foreground_percent": "0.33",
43	"plans_manager": "{'dataset_name': 'Dataset006_TCIA_FeTS002_EGD_SS_N4_BG_Tio_MCon_data', 'plans_name': 'nnUNetPlans', 'original_median_spacing_after_transp': [1.0, 1.0, 1.0], 'original_median_shape_after_transp': [189, 233, 197], 'image_reader_writer': 'SimpleITKIO', 'transpose_forward': [0, 1, 2], 'transpose_backward': [0, 1, 2], 'configurations': {'2d': {'data_identifier': 'nnUNetPlans_2d', 'preprocessor_name': 'DefaultPreprocessor', 'batch_size': 100, 'patch_size': [256, 224], 'median_image_size_in_voxels': [233.0, 197.0], 'spacing': [1.0, 1.0], 'normalization_schemes': ['ZScoreNormalization', 'ZScoreNormalization', 'ZScoreNormalization', 'ZScoreNormalization'], 'use_mask_for_norm': [False, False, False, False], 'UNet_class_name': 'PlainConvUNet', 'UNet_base_num_features': 32, 'n_conv_per_stage_encoder': [2, 2, 2, 2, 2, 2], 'n_conv_per_stage_decoder': [2, 2, 2, 2, 2], 'num_pool_per_axis': [5, 5], 'pool_op_kernel_sizes': [[1, 1], [2, 2], [2, 2], [2, 2], [2, 2], [2, 2]], 'conv_kernel_sizes': [[3, 3], [3, 3], [3, 3], [3, 3], [3, 3], [3, 3]], 'unet_max_num_features': 512, 'resampling_fn_data': 'resample_data_or_seg_to_shape', 'resampling_fn_seg': 'resample_data_or_seg_to_shape', 'resampling_fn_data_kwargs': {'is_seg': False, 'order': 3, 'order_z': 0, 'force_separate_z': None}, 'resampling_fn_seg_kwargs': {'is_seg': True, 'order': 1, 'order_z': 0, 'force_separate_z': None}, 'resampling_fn_probabilities': 'resample_data_or_seg_to_shape', 'resampling_fn_probabilities_kwargs': {'is_seg': False, 'order': 1, 'order_z': 0, 'force_separate_z': None}, 'batch_dice': True}, '3d_fullres': {'data_identifier': 'nnUNetPlans_3d_fullres', 'preprocessor_name': 'DefaultPreprocessor', 'batch_size': 2, 'patch_size': [192, 256, 192], 'median_image_size_in_voxels': [189.0, 233.0, 197.0], 'spacing': [1.0, 1.0, 1.0], 'normalization_schemes': ['ZScoreNormalization', 'ZScoreNormalization', 'ZScoreNormalization', 'ZScoreNormalization'], 'use_mask_for_norm': [False, False, False, False], 'UNet_class_name': 'PlainConvUNet', 'UNet_base_num_features': 32,

	'n_conv_per_stage_encoder': [2, 2, 2, 2, 2, 2], 'n_conv_per_stage_decoder': [2, 2, 2, 2, 2], 'num_pool_per_axis': [5, 5, 5], 'pool_op_kernel_sizes': [[1, 1, 1], [2, 2, 2], [2, 2, 2], [2, 2, 2], [2, 2, 2], [2, 2, 2]], 'conv_kernel_sizes': [[3, 3, 3], [3, 3, 3], [3, 3, 3], [3, 3, 3], [3, 3, 3], [3, 3, 3]], 'unet_max_num_features': 320, 'resampling_fn_data': 'resample_data_or_seg_to_shape', 'resampling_fn_seg': 'resample_data_or_seg_to_shape', 'resampling_fn_data_kwargs': {'is_seg': False, 'order': 3, 'order_z': 0, 'force_separate_z': None}, 'resampling_fn_seg_kwargs': {'is_seg': True, 'order': 1, 'order_z': 0, 'force_separate_z': None}, 'resampling_fn_probabilities': 'resample_data_or_seg_to_shape', 'resampling_fn_probabilities_kwargs': {'is_seg': False, 'order': 1, 'order_z': 0, 'force_separate_z': None}, 'batch_dice': False}}, 'experiment_planner_used': 'ExperimentPlanner', 'label_manager': 'LabelManager', 'foreground_intensity_properties_per_channel': {'0': {'max': 8449.001953125, 'mean': 876.2181396484375, 'median': 587.5035400390625, 'min': -62.86849594116211, 'percentile_00_5': 0.0, 'percentile_99_5': 5023.051276855462, 'std': 860.8373413085938}, '1': {'max': 22296.71484375, 'mean': 1010.2324829101562, 'median': 643.4981079101562, 'min': -217.8645782470703, 'percentile_00_5': 0.0, 'percentile_99_5': 6990.7314453125, 'std': 1197.094970703125}, '2': {'max': 13443.9501953125, 'mean': 906.3700561523438, 'median': 697.4663696289062, 'min': -311.94610595703125, 'percentile_00_5': 0.0, 'percentile_99_5': 4928.394047851558, 'std': 798.8444213867188}, '3': {'max': 11528.87890625, 'mean': 741.3021240234375, 'median': 524.4417724609375, 'min': -217.5197296142578, 'percentile_00_5': 0.0, 'percentile_99_5': 3948.712703857367, 'std': 668.06103515625}}}",
46	"save_every": "50",
47	"torch_version": "2.0.0",
48	"unpack_dataset": "True",
49	"was_initialized": "True",
50	"weight_decay": "3e-05"
51	"_best_ema": "0.8365259113358365",
52	"batch_size": "100",
53	"configuration_manager": "{'data_identifier': 'nnUNetPlans_2d', 'preprocessor_name': 'DefaultPreprocessor', 'batch_size': 100, 'patch_size': [256, 224], 'median_image_size_in_voxels': [233.0, 197.0], 'spacing': [1.0, 1.0], 'normalization_schemes': ['ZScoreNormalization', 'ZScoreNormalization', 'ZScoreNormalization', 'ZScoreNormalization'], 'use_mask_for_norm': [False, False, False, False], 'UNet_class_name': 'PlainConvUNet', 'UNet_base_num_features': 32, 'n_conv_per_stage_encoder': [2, 2, 2, 2, 2, 2], 'n_conv_per_stage_decoder': [2, 2, 2, 2, 2], 'num_pool_per_axis': [5, 5], 'pool_op_kernel_sizes': [[1, 1], [2, 2], [2, 2], [2, 2], [2, 2], [2, 2]], 'conv_kernel_sizes': [[3, 3], [3, 3], [3, 3], [3, 3], [3, 3], [3, 3]], 'unet_max_num_features': 512, 'resampling_fn_data': 'resample_data_or_seg_to_shape', 'resampling_fn_seg': 'resample_data_or_seg_to_shape', 'resampling_fn_data_kwargs': {'is_seg': False, 'order': 3, 'order_z': 0, 'force_separate_z': None}, 'resampling_fn_seg_kwargs': {'is_seg': True, 'order': 1, 'order_z': 0, 'force_separate_z': None}, 'resampling_fn_probabilities': 'resample_data_or_seg_to_shape', 'resampling_fn_probabilities_kwargs': {'is_seg': False, 'order': 1, 'order_z': 0, 'force_separate_z': None}, 'batch_dice': True}",
54	"configuration_name": "2d",
55	"cudnn_version": 8500,
56	"current_epoch": "1250",

57	"dataloader_train": "<nnunetv2.training.data_augmentation.custom_transforms.limited_length_multithreaded_augmenter.LimitedLenWrapper object at 0x2aabf8d86020>",
58	"dataloader_train.generator": "<nnunetv2.training.dataloading.data_loader_2d.nnUNetDataLoader2D object at 0x2aabf8d86b60>",
59	"dataloader_train.num_processes": "100",
60	"dataloader_train.transform": "Compose ([SpatialTransform(independent_scale_for_each_axis = True, p_rot_per_sample = 0.4, p_scale_per_sample = 0.2, p_el_per_sample = 0.2, data_key = 'data', label_key = 'seg', patch_size = [256, 224], patch_center_dist_from_border = None, do_elastic_deform = False, alpha = (0.0, 1000.0), sigma = (10.0, 13.0), do_rotation = True, angle_x = (-3.141592653589793, 3.141592653589793), angle_y = (0, 0), angle_z = (0, 0), do_scale = True, scale = (0.7, 1.43), border_mode_data = 'constant', border_cval_data = 0, order_data = 3, border_mode_seg = 'constant', border_cval_seg = -1, order_seg = 1, random_crop = False, p_rot_per_axis = 0.5, p_independent_scale_per_axis = 1), OneOfTransform(list_of_transforms = [MedianFilterTransform(p_per_sample = 0.2, p_per_channel = 0.5, data_key = 'data', filter_size = (2, 8), same_for_each_channel = False), GaussianBlurTransform(p_per_sample = 0.2, different_sigma_per_channel = True, p_per_channel = 0.5, data_key = 'data', blur_sigma = (0.3, 1.5), different_sigma_per_axis = False, p_isotropic = 0)]), GaussianNoiseTransform(p_per_sample = 0.1, data_key = 'data', noise_variance = (0, 0.1), p_per_channel = 1, per_channel = False), BrightnessTransform(p_per_sample = 0.1, data_key = 'data', mu = 0, sigma = 0.5, per_channel = True, p_per_channel = 0.5), OneOfTransform(list_of_transforms = [ContrastAugmentationTransform(p_per_sample = 0.2, data_key = 'data', contrast_range = (0.5, 2), preserve_range = True, per_channel = True, p_per_channel = 0.5), ContrastAugmentationTransform(p_per_sample = 0.2, data_key = 'data', contrast_range = (0.5, 2), preserve_range = False, per_channel = True, p_per_channel = 0.5)]), SimulateLowResolutionTransform(order_upsample = 3, order_downsample = 0, channels = None, per_channel = True, p_per_channel = 0.5, p_per_sample = 0.15, data_key = 'data', zoom_range = (0.25, 1), ignore_axes = None), GammaTransform(p_per_sample = 0.1, retain_stats = True, per_channel = True, data_key = 'data', gamma_range = (0.7, 1.5), invert_image = True), GammaTransform(p_per_sample = 0.1, retain_stats = True, per_channel = True, data_key = 'data', gamma_range = (0.7, 1.5), invert_image = True), MirrorTransform(p_per_sample = 1, data_key = 'data', label_key = 'seg', axes = (0, 1)), BlankRectangleTransform(rectangle_size = [[25, 85], [22, 74]], num_rectangles = (1, 5), force_square = False, p_per_sample = 0.4, p_per_channel = 0.5, apply_to_keys = ('data',), color_fn = <function BlankRectangleTransform.__init__.<locals>.<lambda> at 0x2aabf4b8b2e0>), <batchgenerators.transforms.local_transforms.BrightnessGradientAdditiveTransform object at 0x2aabf8d85960>, <batchgenerators.transforms.local_transforms.LocalGammaTransform object at 0x2aabf8d85930>, SharpeningTransform(p_per_sample = 0.2, p_per_channel = 0.5, data_key = 'data', strength = (0.1, 1), same_for_each_channel = False), RemoveLabelTransform(output_key = 'seg', input_key = 'seg', replace_with = 0, remove_label = -1), RenameTransform(delete_old = True, out_key = 'target', in_key = 'seg'), DownsampleSegForDSTransform2(axes = None, output_key = 'target', input_key = 'target', order = 0, ds_scales = [[1.0, 1.0], [0.5, 0.5], [0.25, 0.25], [0.125, 0.125], [0.0625, 0.0625]]), NumpyToTensor(keys = ['data', 'target'], cast_to = 'float')])",

61	"dataloader_val": "<nnunetv2.training.data_augmentation.custom_transforms.limited_length_multithreaded_augmenter.LimitedLenWrapper object at 0x2aabf8d849d0>",
